# Interferon-gamma release assay for screening of tuberculosis infection in children

**DOI:** 10.1186/s12879-023-08871-z

**Published:** 2023-12-13

**Authors:** Huiwen Zheng, Jing Xiao, Feina Li, Hao Chen, Deze Li, Yonghong Wang, Yajie Guo, Yuying Chen, Chen Shen

**Affiliations:** 1 Laboratory of Respiratory Diseases, Beijing Key Laboratory of Pediatric Respiratory Infection Diseases, Beijing Children’s Hospital, Key Laboratory of Major Diseases in Children, Ministry of Education, National Clinical Research Center for Respiratory Diseases, Beijing Pediatric Research Institute, Capital Medical University, National Center for Children’s Health, Beijing, 100045 China; 2 Department of Respiratory Medicine, Beijing Children’s Hospital, Capital Medical University, National Center for Children’s Health, Beijing, China

**Keywords:** Latent tuberculosis infection, QFT-GIT, XDOT, Indeterminate, Children

## Abstract

**Background:**

Interferon-gamma release assay (IGRA) is the main tool for the diagnosis of latent tuberculosis (TB) infection (LTBI). However, the indeterminate results were more frequent in children, and the underlying reasons were largely speculative. We aimed to compare QuantiFERON-TB Gold In-Tube (QFT-GIT) with X.DOT-TB (XDOT) for diagnosing LTBI, and to identify the risk factors associated with indeterminate results in children.

**Methods:**

A retrospective study for children<18 years old, at risk for LTBI or progression to TB disease, received either QFT-GIT or X.DOT-TB tests was performed at Beijing Children’s Hospital from August 2019 to August 2022.

**Results:**

A total of 33,662 children were recruited, including 15,129 (44.9%) tested with X.DOT-TB and 18,533 (55.1%) with QFT-GIT. Proportion of positive and indeterminate results in children with respiratory disease was significantly higher than did that with other diseases, respectively (*P* < 0.001). The indeterminate rate of X.DOT-TB and QFT-GIT results decreased with increasing age (*P* < 0.001). Proportion of QFT-GIT indeterminate results was higher than that of X.DOT-TB across age groups. Male, age and disease classification all presented a statistically significant association with indeterminate IGRA results.

**Conclusions:**

The positive rates of X.DOT-TB and QFT-GIT in children were 3.1% and 1.8%, respectively. The X.DOT-TB assay performed better than QFT-GIT in children, and male, age and underlying diseases were associated with an increased risk of indeterminate IGRA results.

**Supplementary Information:**

The online version contains supplementary material available at 10.1186/s12879-023-08871-z.

## Introduction

Tuberculosis (TB) remains a serious threat to public health worldwide. Globally, approximately 10.6 million incident cases were in 2021, including approximately 1.2 million TB cases in children [1]. However, only 10% individuals infected with *Mycobacterium tuberculosis* (MTB) develop clinically active TB disease, and 90% are asymptomatic and remain in the latent phase, constituting a large reservoir of individuals with latent TB infection (LTBI) [2]. Compared to adults, children with LTBI progress more frequently to active TB [3], and the risk rises as high as 50% for children under one year of age, 6–24% between one and five years and 6–12% in adolescents annually [4]. Therefore, early identification and treatment of LTBI is essential to limit the devastating consequences of TB in children.

Immuno-diagnostic tests are the main tools for the diagnosis of LTBI in clinical setting [1, 2]. Though interferon-gamma release assay (IGRA), based on the host immune response against MTB specific antigens, exhibited higher specificity than TST, which had cross-reactions with BCG vaccination [5]. The indeterminate IGRA results have been shown more frequent in children than adults [3], but the underlying reasons are largely speculative. In our hospital, two commercial kits of IGRAs were used clinically, including QuantiFERON-TB Gold In-Tube (QFT-GIT) (QIAGEN, Germany), which measures the concentration of IFN-γ via an Enzyme-linked Immunosorbent Assay (ELISA), and X.DOT-TB (Signature Biotechnology, China), which measures the frequencies of IFN-γ-secreting cells via Enzyme-linked Immunospot Assay (ELISPOT) [6]. Therefore, the aim of this study was to compare QFT-GIT with X.DOT-TB for diagnosing LTBI, and to identify the risk factors associated with indeterminate results in children.

## Methods

### Study design and population

We performed a retrospective study for all children<18 years old, at risk for LTBI or progression to TB disease, received either QFT-GIT or X.DOT-TB tests at Beijing Children’s Hospital, the largest children’s hospital of tertiary facility in China, from August 2019 to August 2022. For the inpatient/outpatient children with IGRA results, we extracted information from their medical records home pages, including medical department and characteristics of the patient (primary diagnosis name, clinically diagnosis name, gender, and age).

### Disease classification

The children were classified according to the clinical diagnosis of diseases. Respiratory diseases mainly include pneumonia, bronchitis, asthma and other related diseases; central nervous system diseases mainly include encephalitis and meningitis; digestive diseases include vomiting, diarrhea, and abdominal pain; urinary diseases mainly include nephrotic syndrome, acute glomerulonephritis, chronic renal failure, IgA nephropathy; hematological/tumor diseases mainly include leukemia, lymphoma, aplastic anemia; rheumatic immune system diseases mainly include henoch-schonlein purpura, systemic lupus erythematosus, juvenile idiopathic arthritis, dermatomyositis, connective tissue disease; cardiovascular system mainly include myocardial damage, vasculitis, vascular malformation, other mainly include fever, spasm, arthralgia.

### X.DOT-TB

PHA was utilized as the positive control, AIM-V as nil control, and ESAT-6 and CFP-10 as specific antigens in X.DOT-TB test. Following X.DOT-TB manufacturer’s instructions, 5 ml of peripheral venous blood obtained from subjects was into heparin lithium-anticoagulant tubes. Peripheral blood mononuclear cells (PBMCs) within 4 h of collection were seeded (2.5 × 10^6^ cell/ml) on a plate precoated with antibody against IFN-*γ*. Plates were incubated for 20–22 h at 37◦C in 5% carbon dioxide. After incubation, a conjugate against the antibody and enzyme-substrate was generated. Spot-forming cells (SFCs) were counted with an automated ELISpot reader (AID-ispot, Strassberg, Germany). The result was interpreted in supplementary Table 1.

### QuantiFERON-TB gold in tube

According to the manufacturer’s instructions, 1 mL of whole blood was collected into each of the three separate test tubes, including a nil tube, a positive tube with mitogen, and a TB antigen tube (containing ESAT-6, CFP-10 and TB7.7), followed by incubation for 16-24 h at 37 °C. The tubes were centrifuged and supernatants were collected to assess the concentration of IFN‐γ (IU/mL) via ELISA. The result was interpreted in supplementary Table 2.

### Statistical analysis

Categorical variables were presented as percentages, while continuous variables were presented as means and standard deviations. *P*-values < 0.05 were considered statistically significant. Multivariable models were built using “Enter” logistic regression procedures. Data analyses were conducted using SPSS version 23.0.

## Results

### Characteristics of study population

**Fig. 1:**
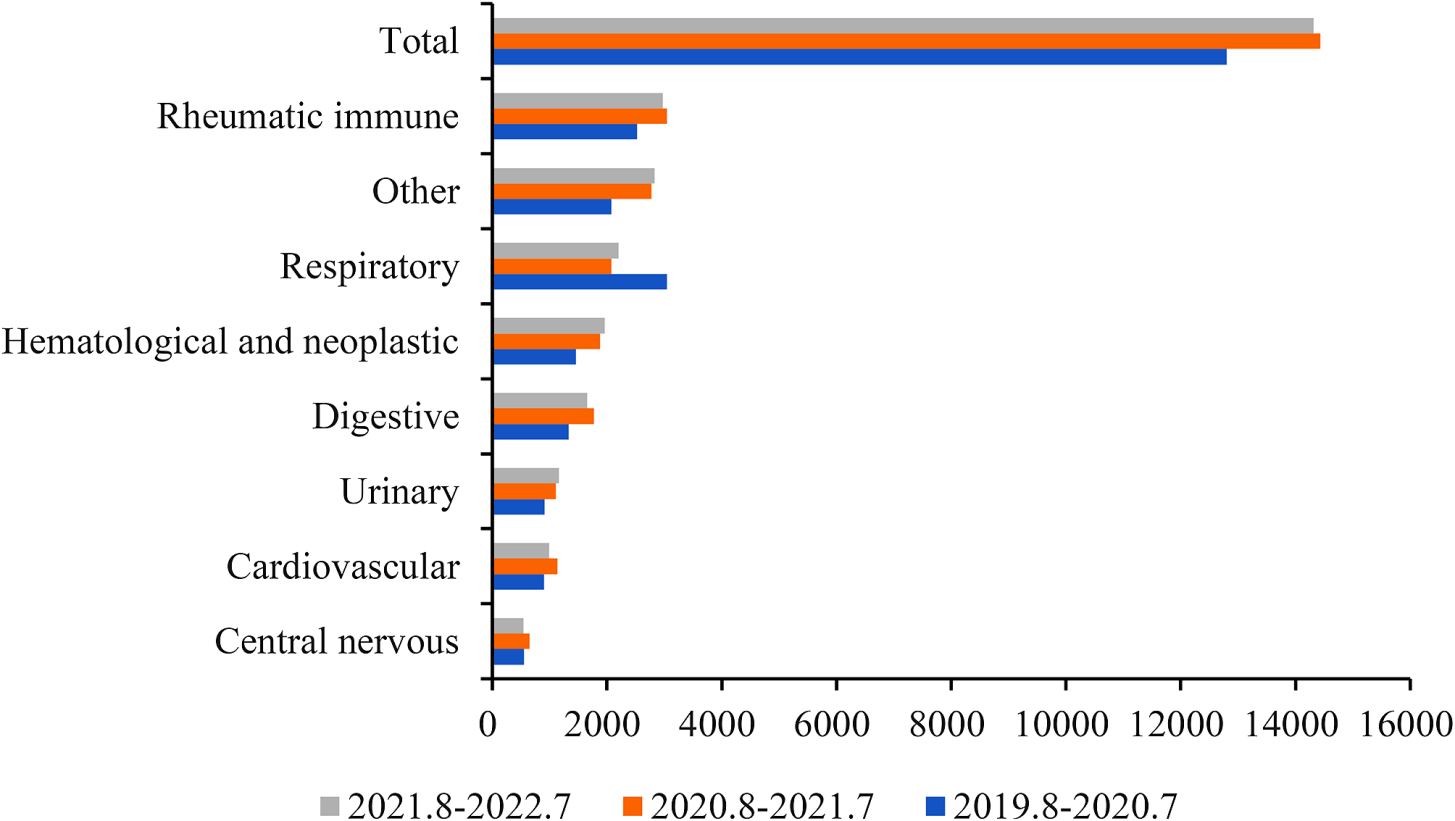
Trends of children with various diseases, 2019 to 2022

**Fig. 2:**
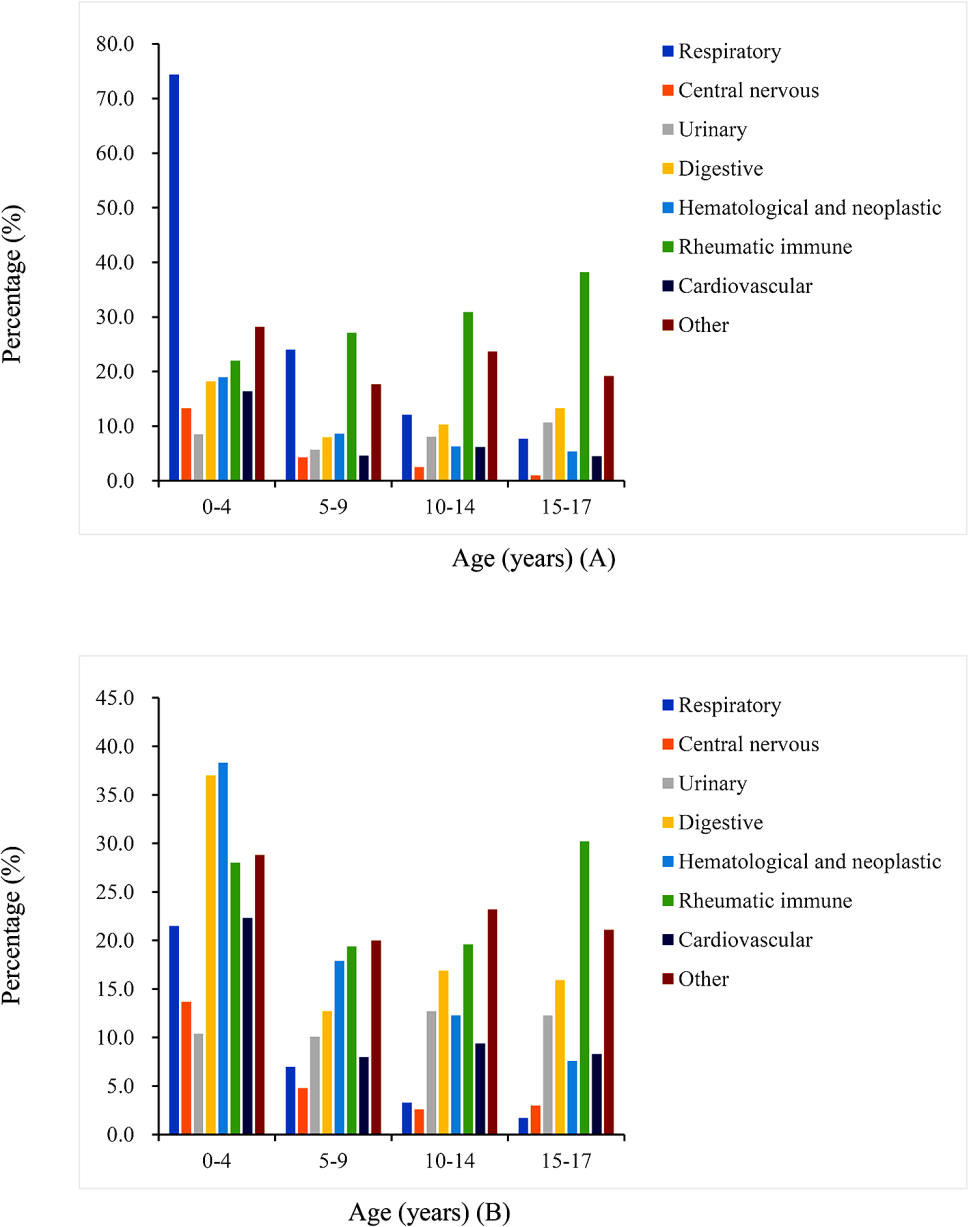
Distribution of various disease among age groups (**A**: children screened by X.DOT-TB; **B**: children screened by QFT-GIT)

A total of 33,662 children screening for LTBI were recruited, including 15,129 (44.9%) tested with X.DOT-TB and 18,533 (55.1%) with QFT-GIT, and 56.1% were male. The mean age was 7.2 years; 33.8% and 33.6% of the patients were aged 5 to 9 and 0 to 4 years, respectively. Children with rheumatic immune (20.5%) and respiratory diseases (17.6%) were more frequently to be screened for LTBI (Table 1). Except for children with respiratory diseases, the number of children with other diseases in 2019 to 2020 year was all lower than that in the other two years, but there was no significant difference (Fig. 1). Besides, for X.DOT-TB assay, larger number of children under 5 years old with respiratory disease and children aged 5 to 17 years with rheumatic immune disease were screened for LTBI. For QFT-GIT, children under 5 years old with hematological and neoplastic disease, and children aged 5 to 17 years with rheumatic immune and other disease were more common to be tested (Fig. 2).

**Table 1:** Baseline characteristics of the study population

Characteristic	X.DOT-TB (*N* = 15,129) *n*(%)	QFT-GIT (*N* = 18,533) *n*(%)	Total (*N* = 33,662) *n*(%)
Gender			
M	8394(55.5)	10,485(56.6)	18,879(56.1)
F	6735(44.5)	8048(43.4)	14,783(43.9)
Age group, y	7.0 ± 4.5	7.4 ± 4.2	7.2 ± 4.3
0–4	5080(33.6)	6039(32.6)	11,119(33)
5–9	5109(33.8)	6419(34.6)	11,528(34.2)
10–14	4314(28.5)	5385(29.1)	9699(28.8)
15–19	626(4.1)	690(3.7)	1316(3.9)
Diseases Classification			
Respiratory	4459(24.0)	2863(12.5)	7322(17.6)
Central nervous	781(4.2)	965(4.2)	1746(4.2)
Urinary	1116(6.0)	2064(9.0)	3180(7.7)
Digestive	1670(9.0)	3096(13.5)	4766(11.5)
Hematological and neoplastic	1558(8.4)	3734(16.2)	5292(12.7)
Rheumatic immune	4433(23.9)	4103(17.8)	8536(20.5)
Cardiovascular	1119(6.0)	1904(8.3)	3023(7.3)
Other	3421(18.4)	4263(18.5)	7684(18.5)

### Results of X.DOT-TB and QFT-GIT assay

Of the 15,129 children tested by X.DOT-TB (Table 2), 472 (3.1%) were positive and 626 (4.1%) were indeterminate. The proportion of positive results in children between 10 and 14 years old (38.8%) was significantly greater than that in other age groups (*P* < 0.001). The indeterminate rate of X.DOT-TB results decreased with increasing age (*P* < 0.001). Concerning disease types, the proportion of positive and indeterminate result in children with respiratory disease was significantly higher than did that with other diseases (*P* < 0.001).

**Table 2:** X.DOT-TB and QFT-GIT Results

Variable	X.DOT-TB				QFT-GIT			
	Total, *n*	Determinate		Indeterminate, *n*(%)	Total, *n*	Determinate		Indeterminate, *n*(%)
		Positive, *n*(%)	Negative, *n*(%)			Positive, *n*(%)	Negative, *n*(%)	
Age (years)								
0–4	5080	93(19.7)	4706(33.5)	281(44.8)	6039	72(21.2)	4627(29.4)	1340(55.4)
5–9	5109	155(32.8)	4759(33.9)	195(31.2)	6419	102(29.9)	5539(35.2)	778(32.1)
10–14	4314	183(38.8)	4003(28.5)	128(20.4)	5385	145(42.5)	4968(31.6)	272(11.2)
15–17	626	41(8.7)	563(4.0)	22(3.5)	690	22(6.5)	638(4.1)	30(1.2)
Total	15129	472	14031	626	18533	341	15722	2420
Department								
E.N.T.	3	0(0.0)	3(0.0)	0(0.0)	6	0(0.0)	6(0.0)	0(0.0)
Rheumatology and immunology	1356	26(5.5)	1277(9.1)	53(8.5)	571	8(2.3)	458(2.9)	105(4.3)
Infection	1514	20(4.2)	1407(10.0)	87(13.9)	520	5(1.5)	433(2.7)	82(3.4)
Orthopedic	9	0(0.0)	8(0.1)	1(0.2)	38	9(2.6)	29(0.2)	0(0.0)
Pulmonary medicine	2981	137(29.0)	2754(19.6)	90(14.4)	1015	42(12.3)	800(5.1)	173(7.1)
Emergency	388	0(0.0)	370(2.6)	18(2.9)	168	1(0.3)	112(0.7)	55(2.3)
Out-patient	665	48(10.2)	603(4.3)	14(2.2)	594	38(11.1)	522(3.3)	34(1.4)
Internal medicine	2346	163(34.5)	2083(14.8)	100(16.0)	2185	126(37.0)	2018(12.8)	41(1.7)
Dermatology	260	5(1.1)	245(1.7)	10(1.6)	745	5(1.5)	727(4.6)	13(0.5)
Neurology	496	2(0.4)	471(3.4)	23(3.7)	1840	15(4.4)	1690(10.7)	135(5.6)
Nephrology	403	8(1.7)	376(2.7)	19(3.0)	1518	23(6.7)	1278(8.1)	217(9.0)
Surgery	7	1(0.2)	4(0.0)	2(0.3)	8	0(0.0)	7(0.0)	1(0.0)
Digestive	489	16(3.4)	448(3.2)	25(4.0)	1914	12(3.5)	1761(11.2)	141(5.8)
Cardiology	810	3(0.6)	780(5.6)	27(4.3)	2484	15(4.4)	1798(11.4)	671(27.7)
Neonatology	19	0(0.0)	16(0.1)	3(0.5)	26	1(0.3)	22(0.1)	3(0.1)
Hematology	674	10(2.1)	629(4.5)	35(5.6)	2930	19(5.6)	2509(15.9)	402(16.6)
Transplantation	52	0(0.0)	52(0.4)	0(0.0)	81	0(0.0)	65(0.4)	16(0.7)
Traditional Chinese Medicine	1976	25(5.3)	1887(13.4)	64(10.2)	940	14(4.1)	822(5.2)	104(4.3)
Oncology	199	1(0.2)	186(1.3)	12(1.9)	232	0(0.0)	195(1.2)	37(1.5)
Intensive care unit	171	1(0.2)	151(1.1)	19(3.0)	92	0(0.0)	51(0.3)	41(1.7)
General	146	1(0.2)	133(0.9)	12(1.9)	457	1(0.3)	318(2.0)	138(5.7)
Other	165	5(1.1)	148(1.1)	12(1.9)	169	7(2.1)	151(1.0)	11(0.5)
Total	15129	472	14031	626	18533	341	15722	2420
Diseases Classification								
Respiratory	4459	145(30.3)	4137(24.0)	177(21.6)	2863	81(14.4)	2097(11.1)	685(19.3)
Central nervous	781	21(4.4)	720(4.2)	40(4.9)	965	13(2.3)	838(4.4)	114(3.2)
Urinary	1116	25(5.2)	1050(6.1)	41(5.0)	2064	31(5.5)	1763(9.3)	270(7.6)
Digestive	1670	49(10.3)	1524(8.8)	97(11.8)	3096	43(7.6)	2580(13.7)	473(13.3)
Hematological and neoplastic	1558	23(4.8)	1415(8.2)	120(14.6)	3734	27(4.8)	3173(16.8)	534(15.1)
Rheumatic immune	4433	86(18.0)	4173(24.2)	174(21.2)	4102	56(9.9)	3079(16.3)	967(27.3)
Cardiovascular	1119	19(4.0)	1053(6.1)	47(5.7)	1904	225(40.0)	1431(7.6)	248(7.0)
Other	3421	110(23.0)	3187(18.5)	124(15.1)	4263	87(15.5)	3922(20.8)	254(7.2)
Total	18557	571	17259	820	22991	563	18883	3545

Of the 18,533 children tested by QFT-GIT (Table 2), 341 (1.8%) were positive and 2420 (13.1%) were indeterminate. The proportion of positive results in children between 10 and 14 years old (42.5%) was significantly greater than that in other age groups (*P* < 0.001). The indeterminate rate of QFT-GIT results also decreased with increasing age (*P* < 0.001). As for disease types, the proportion of positive result in children with cardiovascular disease was significantly higher than that with other diseases (*P* < 0.001), and a higher proportion of indeterminate QFT-GIT result was observed among children with rheumatic disease (*P* < 0.001).

**Fig. 3:**
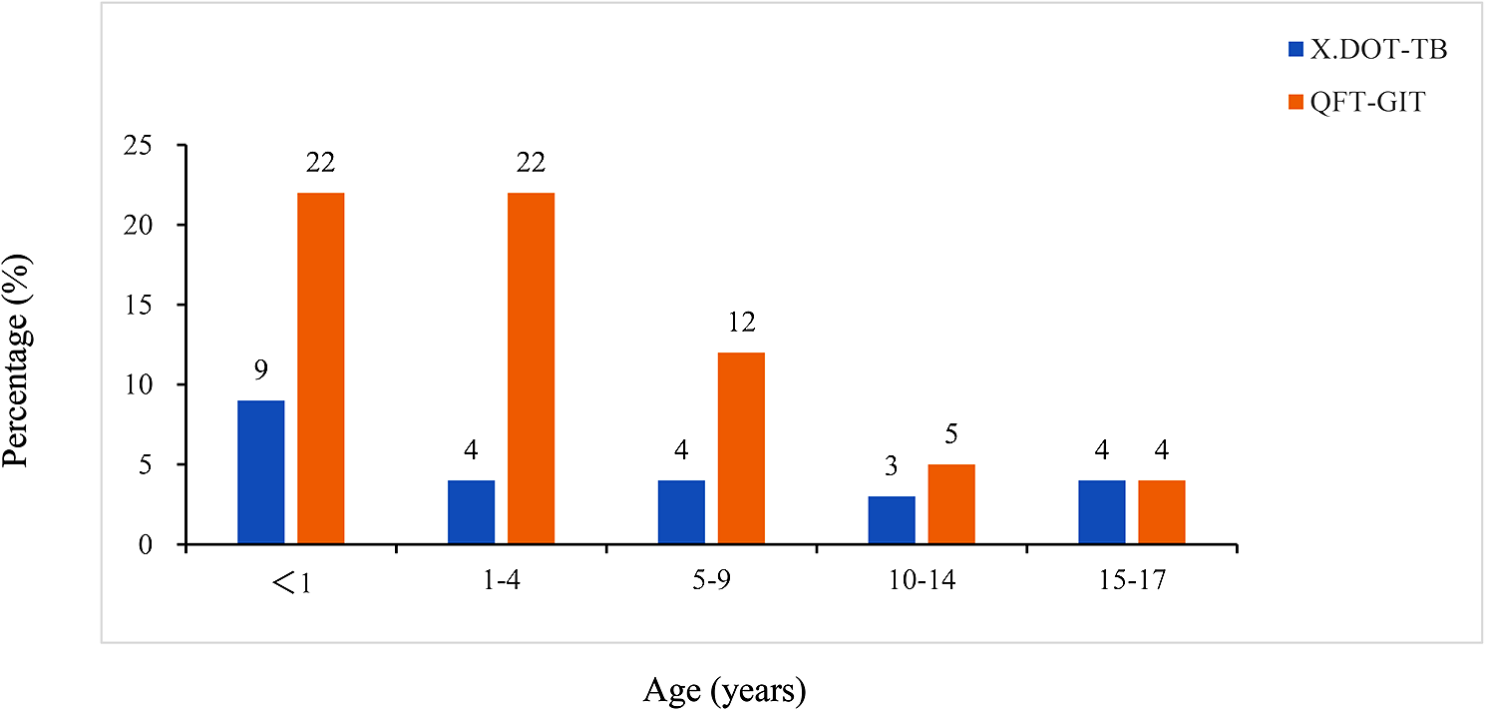
Indeterminate X.DOT-TB and QFT-GIT TB results by Ages

A comparison of indeterminate results for the two methods by age can be found in Fig. 3. The proportion of QFT-GIT indeterminate results was higher than that of X.DOT-TB across age groups.

### Risk factors for indeterminate results

Univariable logistic regression analysis revealed that indeterminate X.DOT-TB results differed across age groups. Using children aged between 15 and 17 as a control group, children under five years old were more likely to have indeterminate result (44.8%) than that with determinate result (33.1%). In addition, children, with digestive diseases (11.8%) and hematological and neoplastic diseases (14.6%), had significantly higher odds of having indeterminate result compared with determinate result (8.9% and 8.1%, respectively). Moreover, multivariate logistic modelling analysis showed that being male (adjusted odds ratio [aOR] 0.71, 95% CI 0.66–0.76), younger than 10 years (aOR 2.04, 95% CI 1.70–2.45 for children under 5 years old; aOR 1.37, 95% CI 1.14–1.64 for children 5–9 years of age), digestive diseases (aOR 1.61, 95% CI 1.43–1.82), hematological and neoplastic diseases (aOR 1.97, 95% CI 1.78–2.19) and rheumatic immune diseases (aOR 1.16, 95% CI 1.06–1.27) had a higher risk of indeterminate X.DOT-TB result (Table 3).

**Table 3:** Multivariate analysis of risk factors associated with Indeterminate X.DOT-TB results in childhood tuberculosis

Variable	Indeterminate (*n* = 626)	Determinate (*n* = 14,503)	Univariable analysis			Multivariate analysis	
			Crude OR (95% CI)	*P* value		Adjusted OR (95% CI)	*P* value
Gender							
M	390(62.3)	8004(55.2)	Reference	-		Reference	-
F	236(37.7)	6499(44.8)	0.75(0.63–0.88)	<0.01		0.71(0.66–0.76)	<0.001
Age group, y							
0–4	281(44.8)	4799(33.1)	1.61(1.03–2.50)	0.03		2.04(1.70–2.45)	<0.001
5–9	195(31.2)	4914(33.9)	1.09(0.70–1.71)	0.71		1.37(1.14–1.64)	0.001
10–14	128(20.4)	4186(28.9)	0.84(0.53–1.33)	0.46		0.90(0.74–1.08)	0.26
15–17	22(3.5)	604(4.2)	Reference	-		Reference	-
Diseases Classification							
Respiratory	177(21.6)	4282(24.1)	1.10(0.87–1.39)	0.43		0.91(0.77–1.07)	0.25
Central nervous	40(4.9)	741(4.2)	1.44(1.00-2.07)	0.05		1.19(0.94–1.50)	0.15
Urinary	41(5.0)	1075(6.1)	1.01(0.71–1.45)	0.94		1.05(0.87–1.23)	0.61
Digestive	97(11.8)	1573(8.9)	1.64(1.25–2.15)	<0.01		1.61(1.43–1.82)	<0.001
Hematological and neoplastic	120(14.6)	1438(8.1)	2.22(1.71–2.87)	<0.01		1.97(1.78–2.19)	<0.001
Rheumatic immune	174(21.2)	4259(24.0)	1.09(0.86–1.37)	0.49		1.16(1.06–1.27)	0.001
Cardiovascular	47(5.7)	1072(6.0)	1.17(0.83–1.64)	0.38		1.06(0.93–1.21)	0.37
Other	124(15.1)	3297(18.6)	Reference	-		Reference	-

Univariable logistic regression analysis revealed that the rate of QFT-GIT indeterminate result for children under five years old (55.4%) was significantly higher than that of determinate result (29.2%). And the percentage of indeterminate result (32.1%) was significantly lower than that of determinate result (35.0%) for children aged 5 to 9. In addition, children with respiratory diseases (19.3%) and rheumatic immune diseases (27.3%) had significantly higher odds of having indeterminate result compared with determinate result (11.2% and 16.1%, respectively). Among the risk factors analyzed through logistic regression, gender, age and disease classification all presented a statistically significant association with an increased risk of obtaining indeterminate QFT-GIT results (Table 4).

**Table 4:** Multivariate analysis of risk factors associated with Indeterminate QFT-GIT results in childhood tuberculosis

Variable	Indeterminate (*n* = 2420)	Determinate (*n* = 16,113)	Univariable analysis			Multivariate analysis	
			Crude OR (95% CI)	*P* value		Adjusted OR(95% CI)	*P* value
Gender							
M	1382(57.1)	9103(56.5)	Reference	-		Reference	-
F	1038(42.9)	7010(43.5)	0.98(0.90–1.06)	0.57		0.97(0.95-1.00)	0.045
Age group, y							
0–4	1340(55.4)	4699(29.2)	6.27(4.33–9.09)	<0.01		8.29(7.51–9.14)	<0.001
5–9	778(32.1)	5641(35.0)	3.03(2.09–4.41)	<0.01		3.32(3.01–3.67)	<0.001
10–14	272(11.2)	5113(31.7)	1.17(0.80–1.72)	0.42		1.16(1.05–1.29)	0.004
15–17	30(1.2)	660(4.1)	Reference	-		Reference	-
Diseases Classification							
Respiratory	685(19.3)	2178(11.2)	4.96(4.26–5.79)	<0.01		3.64(3.32–4.01)	<0.001
Central nervous	114(3.2)	851(4.4)	2.11(1.68–2.67)	<0.01		1.60(1.44–1.78)	<0.001
Urinary	270(7.6)	1794(9.2)	2.38(1.98–2.84)	<0.01		2.84(2.66–3.02)	<0.001
Digestive	473(13.3)	2623(13.5)	2.85(2.43–3.34)	<0.01		2.86(2.72-3.00)	<0.001
Hematological and neoplastic	534(15.1)	3200(16.5)	2.63(2.25–3.08)	<0.01		2.07(1.99–2.17)	<0.001
Rheumatic immune	967(27.3)	3135(16.1)	4.87(4.21–5.63)	<0.01		5.20(5.00-5.41)	<0.001
Cardiovascular	248(7.0)	1656(8.5)	2.36(1.97–2.84)	<0.01		2.20(2.09–2.30)	<0.001
Other	254(7.2)	4009(20.6)	Reference	-		Reference	-

## Discussion

Children living with HIV/AIDS, having a history of exposure to pulmonary TB cases, initiating anti-tumor necrosis factor therapy, receiving organ or hematologic transplantation, and patients with end-stage renal failure are at high risk of TB infection or progressingto active TB disease [7]. So screening for LTBI in this vulnerable population before treatment was necessary. In this study, we found that children under 5 years old with respiratory disease and hematological and neoplastic disease were more frequently to be screened for LTBI. Of which, pneumonia and anaemia was predominant, respectively. For children under 5 years old, acute lower respiratory tract infections predominating with pneumonia are the leading cause of death, which is difficult to distinguish from tuberculosis due to lack of typical clinical symptoms. Besides, physical and intellectual development is at a critical stage for children under 5 years old, but tuberculosis is often accompanied by malnutrition, which leads to anaemia [8]. For children between 5 and 17 years of age, henoch-schönlein purpura (HSP) of rheumatic immune disease was more likely to be screened for LTBI in our study. The immunosuppressive therapy of HSP, the most common form of systemic vasculitis in children, is a risk factor for reactivation of latent infections [9]. So these children were more likely to be screened for LTBI.

We found a positive rate of 3.1% for X.DOT-TB, and 1.8% for QFT-GIT. According to another research by our team, the positive rate of children younger than 18 years old was 5.3% and 2.2% for household contacts and non-household contacts, respectively [10]. The positive rate was 2.5% for participants aged 5–15 years old from a baseline survey in China for QFT-GIT [11]. Besides, consistent with previous studies [12, 13], significant difference in the positivity rate was observed across the age groups between X.DOT-TB and QFT-GIT in our study. The proportion of positive results in children between 10 and 14 years old (38.8% for X.DOT-TB, 42.5% for QFT) was significantly greater than that in other age groups. The reason may be that BCG vaccination protection declines with time, lasting about 10–15 years. Besides, developing immune system, heavy study load, nutritional imbalance, and lacking of physical exercise for children were on this age. Therefore, screening for LTBI before treatment was of important.

The indeterminate results complicated clinical management and increased costs of further diagnostic testing. In this study, male was more likely to have indeterminate results. However, the conclusions from different studies on gender were not consistent [14–16]. Younger age was considered to be associated with an indeterminate IGRA result [17–19]. Studies have shown that children aged <4 years old, frequency of indeterminate results was higher [20, 21]. Besides, earlier studies demonstrated that young age was independently associated with a higher risk of obtaining indeterminate QFT-GIT results [22, 23]. Consistent with previous studies, we found that both the indeterminate rate of X.DOT-TB and QFT-GIT decreased with increasing age. In addition, Kampmann et al. [24] found that IGRA responses were lower in children aged < 5 years than that aged 5 to 15 years. Some authors have proposed that very young children produce, on average, less IFN-γ than older children [25], which may explain the association. And from the multivariate analysis, our findings revealed that children younger than 10 years and children across all ages were considered a risk factor for the indeterminate X.DOT-TB and QFT-GIT results, respectively. However, the proportion of QFT-GIT indeterminate results was higher than that of X.DOT-TB results across age groups. We hypothesized that the lymphocyte adjustment in X.DOT-TB assays may reduce the risk of an indeterminate result, particularly in patients with reduced lymphocyte count, such as HIV infection or immunocompromising conditions associated with lymphopenia [3]. This assumption was confirmed by results from a meta-analysis, showing that low CD4 cell counts increased indeterminate rates of QFT-GIT but not of X.DOT-TB assays [26].

As for the disease classification, a higher proportion of indeterminate X.DOT-TB and QFT-GIT result was found among children with respiratory disease and rheumatic disease, respectively. Previous studies reported that low-dose steroids were used as adjuvant treatments for pneumonia, dyspnea in asthma, and acute respiratory distress syndrome [27]. Nevertheless, even low doses of steroids can significantly impact QFT-GIT results, with the highest OR for the indeterminate results [27]. Previous studies reported that an immunocompromised status contributed to indeterminate QFT-GIT results for children [23]. Besides, the immunosuppressive drugs treated for rheumatic disease can induce lymphopenia or impaired the function T-cells or antigen-presenting cells, which can significantly affect the indeterminate results [28]. In addition, some underlying diseases were found to be associated with the indeterminate IGRA results [29]. We found that digestive diseases, hematological and neoplastic diseases, and rheumatic immune diseases had a higher risk of indeterminate X.DOT-TB result, and diseases in this study all presented a statistically significant association with an increased risk of obtaining indeterminate QFT-GIT results. Given these risks, standard care was required when screening for LTBI in this vulnerable population.

The strength of this study lies in it was conducted in a routine clinical testing item rather than highly controlled conditions, which contributed to evaluate the test performance adequately. Besides, it involved a considerable number of children with various disease classification, contributing to elucidate the performance of IGRA in populations with certain risk factors. However, our study has some limitations. First, possible selection biases may have occurred due to the retrospective and single center sample of this study. Second, only partial clinical information was explored, which may lead to different results. Third, analysis on culture proven TB was not carried out. Finally, although several risk factors had been investigated, the contribution of other factors, such as HIV status, remained unclear and required further investigation.

## Conclusions

In summary, the positive rates of X.DOT-TB and QFT-GIT in children were 3.1% and 1.8%, respectively. The X.DOT-TB assay performed better than QFT-GIT in children, and male, age and underlying diseases were associated with an increased risk of an indeterminate IGRA results.

### Electronic supplementary material

Below is the link to the electronic supplementary material.

**Supplementary Material 1:** The result criteria for QFT-GIT and X.DOT-TB

## Data Availability

Data supporting the results can be found in this paper. The datasets generated during and analyzed during the current study are available from the corresponding author on reasonable request.

## Abbreviations

IGRA: Interferon-gamma release assay
LTBI: Latent TB infection
QFT-GIT: QuantiFERON-TB Gold In-Tube
XDOT: XDOT.TB
TB: Tuberculosis
MTB: *Mycobacterium tuberculosis*
ELISA: Enzyme-linked Immunosorbent Assay
ELISPOT: Enzyme-linked Immunospot Assay
PBMCs: Peripheral blood mononuclear cells
SFCs: Spot-forming cells
aOR: Adjusted odds ratio
