# Supplementary material for: Interferon-gamma release assay for screening of tuberculosis infection in children
Source: BMC Infect Dis. 2023 Dec 13;23:873. doi: 10.1186/s12879-023-08871-z (PMC10717111; doi:10.1186/s12879-023-08871-z)
Supplement: Supplementary file 1 — Supplementary Material 1: The result criteria for QFT-GIT and X.DOT-TB [file 12879_2023_8871_MOESM1_ESM.docx]

Supplementary table 1 The result criteria for X.DOT-TB

| Spot count in nil control | Spot count in positive control | Spot count of test minus nil control | Results |
| --- | --- | --- | --- |
| ≤10 | ≥20 | ≥11 | Positive |
|  |  | ≤10 | Negative |
|  | ≤19 | ≥11 | Positive |
|  |  | ≤10 | Indeterminate |
| 11≤N≤20 | ≥20 | ≥N | Positive |
|  |  | ＜N | Negative |
|  | ≤19 | ≥N | Positive |
|  |  | ＜N | Indeterminate |
| ＞20 | any | any | Indeterminate |

Supplementary table 2 The result criteria for QuantiFERON-TB gold

| Nil (IU/ml) | TB Antigen minus Nil (IU/ml) | Mitogen minus  Nil (IU/ml) | Result |
| --- | --- | --- | --- |
| ≤8 | ＜0.35 | ≥0.5 | Negative |
|  | ≥0.35 and ＜25% of Nil value | ≥0.5 | Negative |
|  | ≥0.35 and ＜25% of Nil value | Any | Positive |
|  | ＜0.35 | ＜0.5 | Indeterminate |
|  | ≥0.35 and ＜25% of Nil value | ＜0.5 | Indeterminate |
| ＞8 | Any | Any | Indeterminate |
